# Association between abdominal obesity and hypertension: analysis of the Peruvian *Demographic Family Health Survey* (2018-2023)

**DOI:** 10.1590/0102-311XEN143724

**Published:** 2025-07-21

**Authors:** Ivett Laveriano-Terreros, Antonio Bernabe-Ortiz

**Affiliations:** 1 Universidad Científica del Sur, Lima, Peru.

**Keywords:** Abdominal Obesity, Waist Circumference, Hypertension, Body Mass Index, Obesidad Abdominal, Circunferencia de la Cintura, Hipertensión, Índice de Masa Corporal, Obesidade Abdominal, Circunferência de Cintura, Hipertensão, Índice de Massa Corporal

## Abstract

The association between waist circumference (WC) and hypertension, and if this association varies by sex or by body mass index (BMI) categories have not deeply been assessed in the American continent. We aimed to evaluate whether an association between abdominal obesity and hypertension exists and if sex and BMI configure effect modifiers of such association. A secondary analysis of the *Peruvian Demographic Family Health Survey* data was conducted. Subjects aged from 20 to 69 years were included. Hypertension, defined by the Eighth Joint National Committee, was chosen as the outcome, whereas abdominal obesity (using WC cutoffs based on the 2001 *National Cholesterol Education Program Adult Treatment Panel III*) was selected as exposure. Poisson regression was used to report prevalence ratios (PR) and 95% confidence intervals (95%CI). Data from 144,156 subjects [mean age 41.4 (SD = 13.4), 54.8% of whom were women] were analyzed. Prevalence of abdominal obesity and hypertension totaled 45.4 and 19.5%, respectively. The adjusted model associated abdominal obesity with greater hypertension prevalence (PR = 1.31; 95%CI: 1.24-1.39). BMI, but nor sex, was an effect modifier of the association. Thus, those obese by BMI and abdominally obese had the strongest association with hypertension (PR = 2.76; 95%CI: 2.58-2.94) than those with normal BMI and no abdominal obesity. Our results evince a positive association between abdominal obesity and hypertension depending on BMI category. Those obese by both BMI and WC had the strongest association with hypertension. Our results suggest that BMI and WC offer useful markers for hypertension.

## Resumen

La asociación entre la circunferencia de la cintura (CC) y la hipertensión arterial, así como la posibilidad de esta asociación varíe según el sexo o las categorías del índice de masa corporal (IMC), no ha sido evaluada en profundidad en el continente americano. Nuestro objetivo fue evaluar si existe una asociación entre la obesidad abdominal y la hipertensión arterial y, sumado a esto, si el sexo y el IMC configuran modificadores del efecto de dicha asociación. Se realizó un análisis secundario de los datos de la *Encuesta Demográfica y de Salud Familiar* de Perú. Se incluyeron sujetos con edades comprendidas entre los 20 y los 69 años. La hipertensión, definida por el Octavo Comité Nacional Conjunto, fue considerada como desenlace, mientras que la obesidad abdominal (utilizando los puntos de corte de CC basados en el *Panel de Tratamiento del Adultos III del Programa Nacional de Educación sobre el Colesterol* de 2001) fue seleccionada como exposición. Se utilizó la regresión de Poisson para informar las razones de prevalencia (RP) y los intervalos de confianza del 95% (IC95%). Se analizaron los datos de 144.156 sujetos [edad media 41,4 (DE = 13,4), de los cuales el 54,8% eran mujeres]. La prevalencia de obesidad abdominal e hipertensión arterial fue de 45,4 y 19,5%, respectivamente. El modelo ajustado asoció la obesidad abdominal con una mayor prevalencia de hipertensión arterial (RP = 1,31; IC 95%: 1.24-1.39). El sexo no fue un modificador del efecto de la asociación, pero el IMC sí. De esta manera, los individuos obesos por IMC y con obesidad abdominal presentaron la asociación más fuerte con la hipertensión arterial (RP = 2,76; IC95%: 2,58-2,94) que aquellos con un IMC normal y sin obesidad abdominal. Nuestros resultados evidencian una asociación positiva entre la obesidad abdominal y la hipertensión en función de la categoría del IMC. Los obesos tanto por IMC como por CC tenían la asociación más fuerte con la hipertensión. Nuestros resultados sugieren que el IMC y la CC ofrecen marcadores útiles para la hipertensión.

## Resumo

A associação entre circunferência da cintura (CC) e hipertensão arterial e a sua possível variação por sexo ou categorias de índice de massa corporal (IMC) para o continente americano carece de uma avaliação profunda. Buscamos avaliar se há uma associação entre obesidade abdominal e hipertensão arterial e se sexo e IMC configuram modificadores de efeito dessa associação por uma análise secundária dos dados da *Pesquisa Demográfica de Saúde Familiar* do Peru. Indivíduos com idade entre 20 e 69 anos foram incluídos neste estudo. A hipertensão, definida pelo Oitavo Comitê Nacional Conjunto, foi escolhida como desfecho, enquanto a obesidade abdominal (usando pontos de corte de CC com base no *Painel III de Tratamento de Adultos do Programa Nacional de Educação em Colesterol*, 2001) foi selecionada como exposição. A regressão de Poisson foi utilizada para relatar razões de prevalência (RP) e intervalos de confiança de 95% (IC95%). Dados de 144.156 indivíduos foram analisados [idade média de 41,4 (DP = 13,4), 54,8% de mulheres]. As prevalências de obesidade abdominal e hipertensão totalizaram 45,4 e 19,5%, respectivamente. O modelo ajustado associou a obesidade abdominal com maior prevalência de hipertensão arterial (RP = 1,31; IC95%: 1,24-1,39). O sexo não configurou um modificador de efeito da associação, mas o IMC sim. Assim, os indivíduos obesos e com obesidade abdominal apresentaram a associação mais forte com a hipertensão arterial (RP = 2,76; IC95%: 2,58-2,94) do que aqueles com IMC normal e sem obesidade abdominal. Nossos resultados evidenciam uma associação positiva entre obesidade abdominal e hipertensão dependendo da categoria de IMC. Aqueles obesos tanto pelo IMC quanto pela CC apresentaram a associação mais forte com a hipertensão. Nossos resultados sugerem que o IMC e a CC oferecem marcadores úteis para hipertensão.

## Introduction

Worldwide, noncommunicable diseases greatly impact public health, of which cardiovascular diseases feature on the top causes of morbidity and mortality ^1^. Thus, every year, 17.9 million deaths (32% of all deaths) in the world occurs due to cardiovascular diseases, and more than 75% of these deaths are from low- and middle-income countries ^2^. Globally, hypertension causes about 8.5 million deaths due to stroke, ischemic heart disease, and other high blood pressure conditions ^3^. According to a study of global trends in the prevalence of hypertension, the number of adults aged from 30 to 79 years with high blood pressure has increased from 650 million to 1.28 billion during the last three decades, and about 50% of these subjects did not know that they had hypertension ^4^. 

Simultaneously, obesity prevalence increased globally, raising public health concerns ^5^. Although several anthropometric markers are used to assess obesity worldwide ^6^, many are difficult to obtain, rendering body mass index (BMI) and waist circumference (WC) as the most used ^7^. These obesity indicators are easy and low-cost to measure at the population level. Many surveys and population-based studies have included them. For example, in Peru, in which about two thirds of the population have overweight ^8^, the *Demographic Family Health Survey* (*Encuesta Demográfica y de Salud Familiar* - ENDES) routinely collects information about these anthropometric indicators. This survey will configure the base of this analysis. 

Previous studies have shown a strong association between markers of obesity and high blood pressure levels. For example, a cross-sectional study conducted in Hungary found a strong association between abdominal obesity and metabolic risk factors (including high blood pressure levels) in subjects without obesity ^9^. Nevertheless, nationally representative data from the Chinese National Stroke Prevention Project reported that BMI, WC, and visceral and body adiposity indices were positively correlated with hypertension, advising the consideration of sex disparities ^10^. A study in Peru reported an increase in the national prevalence of hypertension from 18.7% in 2015 to 20.6% in 2018 ^11^ and that the association between BMI and blood pressure was independent of stratification by demographic variables ^12^. Nevertheless, the association between abdominal obesity and hypertension, and whether this association varies by sex or by BMI categories are yet to be deeply assessed as limited information exists for South American populations. A previous study using the *National Health and Nutrition Examination Survey* reported that both BMI and WC predicted hypertension in the general population (BMI played an important role in hypertension risk in men, and WC, in women) ^13^.

Therefore, this study aimed to evaluate the association between abdominal obesity and hypertension using information from a nationally representative survey. Moreover, we will evaluate whether sex and body mass index configured effect modifiers of the association of interest.

## Materials and methods

### Study design

A secondary analysis was conducted using information from the ENDES. ENDES is an annually population-based cross-sectional study (representative at the national and regional levels) that has been carried out since 1986 by the National Institute of Statistics and Informatics (INEI, acronym in Spanish) ^14^, and which originally focused on sexual and reproductive health and children growth. In 2014, a health questionnaire, body mass index assessment, and blood pressure evaluations were included to it; from 2018 and onward, waist circumference measures were also added. Thus, data from 2018 to 2023 were used for this analysis.

### Study population and sampling

ENDES is a survey whose data framework (i.e., clusters and household selection) is updated every three years, precluding the same participant being selected in the same data framework and reducing such risk between data frameworks. ENDES usually includes all private households and their members, habitual residents, non-residents who spent the night before the day of the interview at the household, and women of childbearing age. Subjects who were aged ≥ 15 years and that could understand the procedures were included in the health questionnaire used for this study ^14^.

For this analysis, information from participants who were aged from 20 to 69 years and who filled out the health questionnaire was analyzed. The exclusion of those who were aged < 20 years and those aged ≥ 70 years was due to the significant variation in blood pressure levels and body weight in these age groups ^15^ ^,^ ^16^. Those with incomplete data on systolic (SBP) and diastolic (DBP) blood pressure, WC, and BMI were also excluded. Extreme blood pressure values either above (SBP >270 mm Hg, DBP > 200mmHg) or below (SBP < 50mmHg, DBP < 50mmHg) average were excluded as in a previous international report ^4^. Finally, information on pregnant women and/or those who were breastfeeding were also excluded.

ENDES sampling follows a two-stage probabilistic approach conducted by region and stratified by urban and rural areas ^14^. In the first stage, for urban clusters, the primary sampling unit comprised blocks and a group of blocks containing an average of 140 households, while the secondary sampling unit comprises a randomly selected private household from the primary sampling unit. On the other hand, in rural areas, the rural cluster comprised one or several previously registered rural population centers, whereas households were randomly selected within the chosen clusters in the second stage.

### Definition of variables

Arterial hypertension was chosen as the outcome variable as defined according to the Eighth Joint National Committee (JNC-8) ^17^ as SBP ≥ 140mmHg or DBP ≥ 90mmHg or as subjects reporting a previous diagnosis or receiving treatment for hypertension. Previous report of hypertension was based on the question: “Have you ever been diagnosed with high blood pressure or hypertension by a physician?” An automatic digital monitor (OMRON M5-i, https://www.omron.com/global/en/) was used to evaluate participants’ blood pressure. In total, two measurements were made at two-minute intervals after five minutes of rest. Their average was used for estimations. To avoid alterations in blood pressure values, evaluations were performed before any anthropometric measurements. Both blood pressure measurements were used for this analysis considering the absence of differences greater than 10mmHg between both evaluations, as in previous studies ^11^.

Abdominal obesity was chosen as the exposure of interest. It was defined based on the 2001 *National Cholesterol Education Program Adult Treatment Panel III* (NCEP ATP III) to define metabolic syndrome: a waist circumference ≥ 102cm for men or ≥ 88cm for women ^18^. Waist circumference was measured using a retractable abdominal measuring tape (0.5cm wide and 2 meters long), with a 0.1cm accuracy. Additionally, a pencil was used to mark the reference points on the participant’s body. In total, four imaginary reference points were marked and a midpoint was then established between the bottom of the rib cage and the top edge of the iliac crest, in which the anthropometric measurement was performed ^19^.

In total, two variables were evaluated as potential effect modifiers of the association between abdominal obesity and hypertension: sex (women vs. men) and BMI (normal, overweight, and obesity). In the case of weight, a SECA brand electronic scale (https://www.seca.com/es_pe/soporte-tecnico/qualitymanagement-mdr.html) was used, and information was collected in kilograms (kg); whereas height was measured by a standard stadiometer in centimeters (cm) ^19^. Traditional cutoffs were used to define overweight (BMI ≥ 25 and < 30kg/m^2^) and obesity (BMI ≥ 30kg/m^2^), according to international references ^20^.

Other variables were included in the analysis as potential confounders following the literature ^12^, including sociodemographic, geographic, and behavioral variables. The following sociodemographic variables were chosen: age in years (< 40, 40-59, and ≥ 60 years); education level in years (< 7, 7-11, and ≥ 12 years, compatible with primary, secondary, and superior education, respectively), and socioeconomic level based on the ENDES wealth index (using a standard methodology and easy-to-collect data on owned selected assets in a household, such as televisions and bicycles; materials used to build the house; and types of water access and sanitation facilities) ^21^, split into tertiles (low, medium, high). On the other hand, of the geographical variables, study area (rural vs. urban) and altitude [≤ 500, 501-2,500, ≥ 2,501 meters above sea level (m.a.s.l.)] were added. Finally, some lifestyle variables were included, such as smoking, defined as self-reported consumption of at least one cigarette in the previous month (yes vs. no), and alcohol consumption, defined as self-reported consumption of any alcohol in the previous month (yes vs. no).

### Procedures

Data collection was based on the ENDES protocol using a standard methodology ^14^ ^,^ ^19^. All participants were informed about the collection process and the anthropometric and blood pressure measurements before signing an informed consent form. Fieldwork activities were conducted by trained teams. Information was collected using tablet computers. In 2020, due to the COVID-19 pandemic, activities were modified according to restrictions imposed by the Peruvian government. In that year, data were collected by telephone interviews, and anthropometric evaluations were delayed and carried out by a biosafety protocol after governmental restrictions were reduced.

### Statistical analysis

Stata 16 for Windows (https://www.stata.com) was used for statistical analysis. All analyses were carried out considering the multistage design of the surveys (primary and secondary sampling units and sampling weights) using the *svy* command and the options for subpopulation management ^22^.

Initially, the study population was described based on abdominal obesity and hypertension status. Numerical variables were described using summary measures (mean) and dispersion (standard deviation [SD]), whereas frequency and proportions were used for qualitative variables. To compare the categorical variables of interest, the chi-squared test with the Rao-Scott second-order correction was used ^23^. To evaluate the association between variables of interest, crude and adjusted Poisson regressions were used, reporting prevalence ratios (PR) and 95% confidence intervals (95%CI). The adjusted models included variables reported in the literature as potential confounders for the association of interest. Study year was also included to account for potential changes in conditions over time (i.e., such as the reduction of sample size due to the COVID-19 pandemic in 2020), and BMI was added to evaluate the independent effect of abdominal obesity on hypertension prevalence.

Finally, two approaches were used to assess effect modification. In the first one, the relevance of the effect modifiers, sex, and BMI was evaluated using the Wald test and considering a p-value < 0.05 as significant. When sex was assessed as effect modifier, BMI was included as confounder in the models (similarly, sex was included as confounder when BMI was assessed as effect modifier). However, given the importance of BMI crude and adjusted models were included in the second approach to evaluate a cross-classification of body mass index and abdominal obesity as a single variable. Thus, estimates were calculated for the combinations of BMI and WC subgroups, using the normal BMI and no abdominal obesity as the reference category. In addition to the overall analyses using this second approach, results were stratified by sex.

### Ethical statement

Anonymous and freely available data on the ENDES website were used (https://proyectos.inei.gob.pe/microdatos/). The INEI used informed consent to enroll participants in accordance with the *Declaration of Helsinki*. The database has no personal identifiers to guarantee participants’ confidentiality and anonymity. This study was reviewed and approved by the Research Ethics Committee at Universidad Científica del Sur, Lima, Peru (code: 560-2021-PRE15).

## Results

### Characteristics of the study population

A total of 225,358 subjects participated in the ENDES annual evaluations from 2018 to 2023. Of them, 54,061 (23.9%) stood outside the age range for analysis (< 20 years or ≥ 70 years); 2,219 (1%) women were pregnant or breastfeeding at the moment of this study; 22,960 (10.2%) lacked blood pressure information, and 1,962 (0.9%) lacked abdominal circumference or BMI data. Thus, a total of 81,202 (36%) participants were excluded from this analysis ((Table S1 compares those included and excluded in the analysis. Supplementary Material; https://cadernos.ensp.fiocruz.br/static//arquivo/suppl-e00143724_1108.pdf)). Finally, this study analyzed data from 144,156 (64%) subjects, mean age of 41.4 (SD = 13.4) and of which 82,123 (54.8%) were women.

### Abdominal obesity and associated factors

Mean waist circumference was 93.2 (SD = 11.4) cm, resulting in a prevalence of abdominal obesity of 45.4% (95%CI: 44.9-45.9). When comparing by sex, the mean WC totaled 92.7 (SD = 12.0) in women and 93.8 (SD = 11.6) in men, resulting in a prevalence of abdominal obesity of 22.0% (95%CI: 21.4-22.7) in men and 64.7% (95%CI: 64.1-65.3) in women, respectively. Sex, age, educational level, socioeconomic level, study area, altitude, smoking, alcohol consumption, BMI, and study year were associated with abdominal obesity (Table 1).

**Table 1:** Description of the study population by abdominal obesity.

Characteristic	Abdominal obesity		p-value *
	No [n = 80,492]	Yes [n = 63,664]	
	n (%)	n (%)	
Sex			< 0.001
Female	30,048 (35.5)	52,075 (78.1)	
Male	50,444 (64.5)	11,589 (21.9)	
Age (years)			< 0.001
< 40	52,256 (57.7)	34,782 (39.6 )	
40-59	21,817 (31.5)	22,461 (44.7)	
≥ 60	6,419 (10.8)	6,421 (15.7)	
Education level (years)			< 0.001
< 7	18,184 (19.7)	15,771 (24.2)	
7-11	34,956 (41.1)	26,836 (41.7)	
≥ 12	26,007 (39.2)	20,063 (34.1)	
Socioeconomic level			< 0.001
Low	30,845 (26.6)	16,161 (16.8)	
Middle	26,254 (30.2)	23,416 (32.4)	
High	23,393 (43.2)	24,087 (50.8)	
Study area			< 0.001
Rural	31,744 (26.1)	17,581 (17.4)	
Urban	48,748 (73.9)	46,083 (82.6)	
Altitude (m.a.s.l.)			< 0.001
≤ 500	37,317 (59.3)	34,348 (65.8)	
501-2,500	16,309 (15.7)	12,856 (14.4)	
≥ 2,501	26,866 (25.0)	16,460 (19.8)	
Smoking			< 0.001
No	70,159 (86.8)	59,679 (92.9)	
Yes	10,318 (13.2)	3,968 (7.1)	
Alcohol use			< 0.001
No	51,507 (61.0)	43,954 (66.6)	
Yes	28,954 (39.0)	19,681 (33.4)	
BMI			< 0.001
Normal	43,530 (52.4)	2,350 (3.4)	
Overweight	33,480 (43.0)	27,003 (41.0)	
Obesity	3,482 (4.6)	34,311 (55.6)	
Study year			0.003
2018	14,779 (18.2)	11,692(18.2)	
2019	14,261 (17.7)	11,147 (17.6)	
2020	9,606 (12.3)	7,504 (11.8)	
2021	13,813 (17.2)	11,394 (17.9)	
2022	13,827 (16.9)	11,257 (17.9)	
2023	14,206 (17.7)	10,670 (16.6)	

BMI: body mass index; m.a.s.l.: meters above sea level.

* p-value was estimated using the chi-squared test.

### Hypertension and associated factors

The mean SBP and DBP were 120.7mmHg (SD = 16.9) and 74.9mmHg (SD = 10.3), respectively, with a 19.5% prevalence of hypertension (95%CI: 19.1-19.9). Men showed higher hypertension prevalence (23%; 95%CI: 22.4-23.7) than women (16.6%; 95%CI: 16.1-17.1). Sex, age, educational level, socioeconomic level, area, altitude, alcohol use, BMI, and study year (but not smoking) were associated with hypertension (Table 2).

**Table 2:** Description of the study population by hypertension status.

Characteristic	Hypertension		p-value *
	No [n = 122,837]	Yes [n = 21,319]	
	n (%)	n (%)	
Sex			< 0.001
Female	71,990 (56.8)	10,133 (46.7)	
Male	50,847 (43.2)	11,186 (53.3)	
Age (years)			< 0.001
< 40	80,411 (55.8)	6,627 (23.3)	
40-59	34,645 (35.1)	9,633 (47.6)	
≥ 60	7,781 (9.1)	5,059 (29.1)	
Education level (years)			< 0.001
< 7	27,695 (20.8)	6,260 (25.3)	
7 -11	53,424 (41.5)	8,368 (41.0)	
≥ 12	40,022 (37.7)	6,048 (33.7)	
Socioeconomic level			< 0.001
Low	40,824 (23.1)	6,182 (18.2)	
Middle	42,620 (31.6)	7,050 (29.7)	
High	39,393(45.3)	8,087 (52.1)	
Study area			< 0.001
Rural	42,670 (23.2)	6,655 (18.2)	
Urban	80,167 (76.8)	14,664 (81.8)	
Altitude (m.a.s.l.)			< 0.001
≤ 500	60,099 (61.0)	11,566 (67.6)	
501-2,500	25,047 (15.5)	4,118 (13.6)	
≥ 2,501	37,691 (23.5)	5,635 (18.8)	
Smoking			0.54
No	110,867 (89.5)	18,971 (89.8)	
Yes	11,944 (10.5)	2,342 (10.2)	
Alcohol use			< 0.001
No	81,481 (63.1)	13,980 (65.5)	
Yes	41,306 (36.9)	7,329 (34.5)	
BMI			< 0.001
Normal	42,052 (33.5)	3,828 (16.1)	
Overweight	51,854 (42.4)	8,629 (40.8)	
Obesity	28,931 (24.1)	8,862 (43.0)	
Study year			< 0.001
2018	22,512 (18.4)	3,959 (17.4)	
2019	21,886 (18.1)	3,522 (15.7)	
2020	14,433 (12.0)	2,677 (12.4)	
2021	21,411 (17.1)	3,796 (19.4)	
2022	21,150 (16.9)	3,934 (19.2)	
2023	21,445 (17.5)	3,431 (15.8)	
Abdominal obesity			< 0.001
No	71,372 (58.1)	9,120 (40.1)	
Yes	51,465 (41.9)	12,199 (59.9)	

BMI: body mass index; m.a.s.l.: meters above sea level

* p-value was estimated using the chi-squared test.

### Association between abdominal obesity and hypertension

In the multivariable model − controlling for sex, age, education level, socioeconomic level, study area, altitude, smoking, alcohol use, BMI, and study year − abdominal obesity was associated with a higher hypertension prevalence (PR = 1.31; 95%CI: 1.24-1.39; Table 3). Body mass index (Wald test, p = 0.004) [but not sex (Wald test, p = 0.10)] configured an effect modifier of the association between abdominal obesity and hypertension. By sex, the strength of association was only slightly higher in women (PR = 1.48; 95%CI: 1.33-1.65) than in men (PR = 1.33; 95%CI: 1.23-1.44). On the other hand, by BMI, the association between abdominal obesity and hypertension was greater among those with normal BMI (PR = 1.47; 95%CI: 1.20-1.79) than those with overweight (PR = 1.40; 95%CI: 1.30-1.51) and those with obesity (PR = 1.24; 95%CI: 1.11-1.39). Repeating the analysis only considering those without previous diagnosis of hypertension obtained very similar estimates (Supplementary Material − Table S2; https://cadernos.ensp.fiocruz.br/static//arquivo/suppl-e00143724_1108.pdf).

**Table 3:** Association between abdominal obesity and hypertension: crude and adjusted overall results and stratified by sex and body mass index (BMI).

	Crude mode	Adjusted model *
	PR (95%CI)	PR (95%CI)
Abdominal obesity		
No	1.00 (Reference)	1.00 (Reference)
Yes	1.79 (1.72-1.86)	1.31 (1.24-1.39)
Stratified by sex		
Females		
Without abdominal obesity	1.00 (Reference)	1.00 (Reference)
With abdominal obesity	2.67 (2.48-2.89)	1.48 (1.33-1.65)
Males		
Without abdominal obesity	1.00 (Reference)	1.00 (Reference)
With abdominal obesity	2.31 (2.19-2.44)	1.33 (1.23-1.44)
Stratified by BMI		
Normal		
Without abdominal obesity	1.00 (Reference)	1.00 (Reference)
With abdominal obesity	1.70 (1.43-2.02)	1.47 (1.20-1.79)
Overweight		
Without abdominal obesity	1.00 (Reference)	1.00 (Reference)
With abdominal obesity	1.09 (1.02-1.16)	1.40 (1.30-1.51)
Obesity		
Without abdominal obesity	1.00 (Reference)	1.00 (Reference)
With abdominal obesity	1.13 (1.01-1.27)	1.24 (1.11-1.39)

95%CI: 95% confidence interval; PR: prevalence ratio.

Note: bolded estimates are statistically significant at the 0.05 level.

* Model adjusted by sex, age, education level, socioeconomic level, study area, altitude, smoking, alcohol use, body mass index, and study year. When model was stratified by sex (or BMI), such variable was not included as confounder.

Finally, in the multivariable model, those obese by BMI and abdominally obese had the strongest association with hypertension (PR = 2.76; 95%CI: 2.58-2.94) than those with normal BMI and no abdominal obesity, whereas the lowest association occurred in individuals with overweight by BMI and no abdominal obesity (Table 4). In the analysis stratified by sex, among women, the association with hypertension was significant in cases of abdominal obesity, regardless of BMI category. For example, women with obesity by BMI (but not abdominal obesity) had an insignificant association with hypertension (PR = 1.52; 95%CI: 0.77-3.00) when compared to those with abdominal obesity (PR = 2.41; 95%CI: 2.19-2.67). On the other hand, in the case of men, the association occurred for all the levels of the cross-classification variable, although the strong association occurred in men with normal BMI and abdominal obesity (Table 4). Excluding those with previous diagnosis of hypertension obtained a quite similar relationship (Supplementary Material − Table S3; https://cadernos.ensp.fiocruz.br/static//arquivo/suppl-e00143724_1108.pdf).

**Table 4:** Cross-classification between body mass index (BMI) and abdominal obesity and its association with hypertension, crude and adjusted models.

	Crude mode	Adjusted model *
	PR (95%CI)	PR (95%CI)
BMI and abdominal obesity (overall)		
BMI normal, no abdominal obesity	1.00 (Reference)	1.00 (Reference)
BMI normal, abdominal obesity	1.70 (1.43-2.02)	1.73 (1.44-2.08)
BMI overweight, no abdominal obesity	1.80 (1.68-1.93)	1.55 (1.45-1.66)
BMI overweight, abdominal obesity	1.96 (1.83-2.11)	2.02 (1.88-2.18)
BMI obese, no abdominal obesity	2.67 (2.37-3.02)	2.27 (2.01-2.55)
BMI obese, abdominal obesity	3.03 (2.86-3.22)	2.76 (2.58-2.94)
BMI and abdominal obesity (among women)		
BMI normal, no abdominal obesity	1.00 (Reference)	1.00 (Reference)
BMI normal, abdominal obesity	2.14 (1.77-2.60)	1.42 (1.16-1.74)
BMI overweight, no abdominal obesity	1.07 (0.93-1.24)	1.15 (0.99-1.33)
BMI overweight, abdominal obesity	2.24 (2.02-2.48)	1.73 (1.56-1.92)
BMI obese, no abdominal obesity	1.37 (0.73-2.60)	1.52 (0.77-3.00)
BMI obese, abdominal obesity	3.27 (2.97-3.60)	2.41 (2.19-2.67)
BMI and abdominal obesity (among men)		
BMI normal, no abdominal obesity	1.00 (Reference)	1.00 (Reference)
BMI normal, abdominal obesity	4.97 (2.49-9.92)	3.54 (1.95-6.45)
BMI overweight, no abdominal obesity	1.76 (1.62-1.90)	1.67 (1.54-1.81)
BMI overweight, abdominal obesity	3.25 (2.89-3.66)	2.33 (2.07-2.62)
BMI obese, no abdominal obesity	2.29 (2.01-2.60)	2.31 (2.03-2.63)
BMI obese, abdominal obesity	3.43 (3.17-3.72)	2.87 (2.63-3.13)

95%CI: 95% confidence interval; PR: prevalence ratio.

Note: bolded estimates are statistically significant at the 0.05 level.

* Model adjusted by sex, age, education level, socioeconomic level, study area, altitude, smoking, alcohol use, and study year.

## Discussion

Using a nationally representative sample of adults in Peru, this study evidenced a strong association between abdominal obesity and hypertension. BMI (but not sex) constituted an effect modifier of the association of interest: those with normal BMI had a greater association with hypertension than those with overweight or obesity, an association that differed by sex, being greater in men than women. Thus, women classified as abdominally obese and obese by BMI showed the highest probability of hypertension. On the other hand, men with normal BMI and abdominal obesity had a stronger association. Excluding those with previous hypertension diagnoses obtained consistent, although slightly stronger, results.

There are many studies reporting the association between abdominal obesity and hypertension. For example, a systematic review of prospective cohort studies including data from 2.3 million participants worldwide reported a relative risk of 1.27 (95%CI: 1.15-1.39) for every 10-cm increase in wc, implying that central adiposity play an important role in the development of hypertension ^24^. A systematic review of 10 cross-sectional studies with samples from Indonesia reported similar results ^25^. Large waist circumference increased the risk of developing hypertension, type 2 diabetes, dyslipidemia, and hyperuricemia. The literature has described that abdominal obesity (WC) predominates over other cardiovascular risk factors in the case of metabolic syndrome in Brazilian women ^26^, highlighting the role of abdominal obesity on hyperglycemia and high blood pressure. Another systematic review of potential factors associated with abdominal obesity in Ethiopia reported the relationship between hypertension and a high prevalence of abdominal obesity ^27^. A longitudinal study in Peru using information from three different population groups (i.e., rural, urban, and rural-urban migrants) reported that obesity − evaluated by different anthropometric markers − was associated with hypertension ^28^. Nevertheless, other obesity markers, such as skinfold thickness, were more relevant than abdominal obesity in that study, showing a greater attributable risk than BMI. Moreover, cross-sectional studies, especially those carried out in Asian countries, such as Vietnam ^29^, South Korea ^30^, and China ^31^, reported similar results but estimates demonstrated greater associations, whereas the strength of the association in our study resembled that in studies conducted in populations from Brazil ^32^ and Ethiopia ^33^. Nevertheless, our study shows similar results to the literature.

In this study, sex was not an effect modifier after we controlled our model by several confounders, including BMI. A study using a nationally representative US adult sample reported that BMI and WC were relevant predictors of hypertension. However, BMI played an important role in men, whereas WC was the most important factor in women ^13^. On the contrary, a report of a nationally representative sample of middle-aged and older Chinese adults reported that WC offered the strongest predictor of hypertension for men, but it was suboptimal for women. These contradicting findings highlight the need to better understand the influence of ethnic origin on such association. On the other hand, BMI constituted an effect modifier of the association between abdominal obesity and hypertension: the association was slightly greater in those with normal BMI values than in those with overweight and obesity by BMI. In line with that, estimates were similar in those without previous diagnoses of hypertension (sensitivity analysis: Supplementary Material − Table S2; https://cadernos.ensp.fiocruz.br/static//arquivo/suppl-e00143724_1108.pdf). As in our study, a report using the U.S. National Health and Nutrition Examination Survey found that abdominal obesity was independently associated with hypertension in a multivariable model and controlling by BMI ^34^, although estimates were lower in our case. Moreover, those subjects classified as abdominally obese and with normal BMI, as abdominally obese and overweight, and abdominally obese and obese by BMI showed a progressive increment in their probability of hypertension when compared with individuals who had a normal BMI and no abdominal obesity. Similar results have been reported using the *China Health and Nutrition Survey* according to WC and other adiposity markers ^35^. Although these studies were conducted at the population level as ours, their age groups, WC cutoffs, and nutritional transition phase differed across countries. Despite these apparently counterintuitive findings, evaluating the role of each anthropometric marker in our BMI-and-WC combined variable by sex evinces their usefulness: BMI may not play a role in the increase of hypertension prevalence when compared to waist circumference in women, but both variables are associated with hypertension in men. Moreover, those with normal BMI and abdominal obesity had the strongest association with the chosen outcome. These results may be relevant as men with normal BMI may have excess adiposity that is detectable by abdominal obesity, highlighting the need of including WC in the anthropometric evaluation of excess of weight. 

As in other Latin American countries, obesity and hypertension trends in Peru are increasing ^11^ ^,^ ^36^. WC has been found as a predictor of visceral fat. Its ease of measure and interpretation suggest its use as a strategy to detect cases of hypertension ^37^. Finding the relationship between abdominal obesity and hypertension in our study may provide an additional substrate for cardiovascular risk stratification and guide interventions to reduce cardiovascular morbidity and mortality.

Our study shows that those with normal BMI and abdominal obesity may have increased risk of hypertension, a consistent finding in sensitivity analyses. Since only BMI may not be sufficient for suspecting hypertension, adding waist circumference should offer a good strategy to evaluate high blood pressure. For example, the United States Preventive Services Task Force recommends screening for high blood pressure in adults aged ≥ 18 years ^38^. The screening interval is annual for those with overweight or obesity, but the recommendation does not include those with high WC. The results of our research may contribute to promoting new strategies for early hypertension diagnosis, especially in resource-constrained settings.

The use of a large dataset from a nationally representative study constitutes a relevant strength of this study. Moreover, the use of standardized procedures also constitutes a strength. However, some limitations deserve discussion. Firstly, the cross-sectional nature of this survey can only estimate association (rather than causality). Moreover, temporality can be an issue. However, the analysis excluding those with previous diagnosis of hypertension precludes reverse causality. Secondly, given this is a secondary analysis, the ENDES health questionnaire included no confounders or effect modifier variables in the analysis, such as physical activity or diet patterns (or menopause status in the case of women). Despite that, our results agree with previous reports. Thirdly, there may exist recall bias, especially in self-reported variables such as anti-hypertensive treatment or smoking and alcohol use. Moreover, these variables, especially smoking, were superficially evaluated as information was unavailable. Fourth, some participants may feature in several ENDES surveys, which may have correlated some of their information. However, given the use of three-year periods for data frameworks, we believe this option is almost negligible. Finally, the information collected in 2020, due to the COVID-19 pandemic, may be different for those in the other years as approaches used for data collection were modified.

## Conclusions

Our study demonstrates a positive association between abdominal obesity and hypertension, which was slightly higher in women than men and in those with normal BMI when compared to those with overweight and obesity. Obese individuals by both BMI and WC had the strongest association with hypertension. Our results suggest that both BMI and WC may offer useful risk markers for hypertension and could guide hypertension screening.
