# Supplementary material for: Association between abdominal obesity and hypertension: analysis of the Peruvian Demographic Family Health Survey (2018-2023)
Source: Cad Saude Publica. 2025 Jul 21;41(7):e00143724. doi: 10.1590/0102-311XEN143724 (PMC12337699; doi:10.1590/0102-311XEN143724)
Supplement: Supplementary file 1 [file 1678-4464-csp-41-07-EN143724-s.pdf]

Supplementary Material

Table S1 Description of the study population by inclusion in the analysis.

Characteristic	Not included [n = 81,202] n (%)	Included [n = 144,156] n (%)
**Sex**		
Female	34,056 (59.4)	82,123 (57.0)
Male	23,245 (40.6)	62,033 (43.0)
**Age (years)**		
< 40	34,205 (58.4)	87,038 (60.4)
40-59	8,570 (14.7)	44,278 (30.7)
≥ 60	15,741 (26.9)	12,840 (8.9)
**Education level (years)**		
< 7	14,403 (26.2)	33,955 (23.9)
7-11	28,724 (52.2)	61,792 (43.6)
≥ 12	11,913 (21.6)	46,070 (32.5)
**Socioeconomic level**		
Low	22,232 (35.0)	47,006 (32.6)
Middle	19,568 (30.8)	49,670 (34.5)
High	21,758 (34.2)	47,480 (32.9)
**Study area**		
Rural	26,927 (33.2)	49,325 (34.2)
Urban	54,275 (66.8)	94,831 (65.8)
**Altitude (m.a.s.l.)**		
≤ 500	41,078 (50.6)	71,665 (49.7)
501-2,500	15,234 (18.8)	29,165 (20.2)
≥ 2,501	24,890 (30.6)	43,326 (30.1)
**Smoking**		
No	50,068 (94.5)	129,838 (90.1)
Yes	2,894 (5.5)	14,286 (9.9)
**Alcohol use**		
No	43,201 (81.6)	95,461 (66.3)
Yes	9,731 (18.4)	48,635 (33.7)
**Body mass index**		
Normal	23,699 (54.8)	45,880 (31.8)
Overweight	12,845 (29.7)	60,483 (42.0)
Obesity	6,703 (15.5)	37,793 (26.2)
**Study year**		
2018	11,015 (13.6)	26,471(18.4)
2019	12,066 (14.9)	25,408 (17.6)
2020	20,973 (25.8)	17,110 (11.9)
2021	12,272 (15.1)	25,207 (17.5)
2022	12,266 (15.1)	25,084 (17.4)
2023	12,610 (15.5)	24,876 (17.2)

a.m.a.s.l.: meters above sea level.

Table S2 Association between abdominal obesity and hypertension: crude and adjusted overall results and stratified by sex and body mass index (BMI) excluding those with previous hypertension diagnosis.

	**Crude model RP (95%CI)**	**Adjusted model * RP (95%CI)**
Abdominal obesity		
No	1.00 (Reference)	1.00 (Reference)
Yes	**1.48 (1.40-1.57)**	**1.29 (1.18-1.41)**
**Stratified by sex**		
Females		
Without abdominal obesity	1.00 (Reference)	1.00 (Reference)
With abdominal obesity	**2.90 (2.53-3.32)**	**1.58 (1.30-1.92)**
Males		
Without abdominal obesity	1.00 (Reference)	1.00 (Reference)
With abdominal obesity	**2.44 (2.28-2.61)**	**1.29 (1.16-1.44)**
**Stratified by BMI**		
Normal		
Without abdominal obesity	1.00 (Reference)	1.00 (Reference)
With abdominal obesity	1.27 (0.92-1.75)	**1.66 (1.14-2.40)**
Overweight		
Without abdominal obesity	1.00 (Reference)	1 (Reference)
With abdominal obesity	**0.72 (0.65-0.79)**	**1.38 (1.23-1.55)**
Obesity		
Without abdominal obesity	1.00 (Reference)	1.00 (Reference)
With abdominal obesity	**0.83 (0.72-0.95)**	**1.25 (1.09-1.44)**

95%CI: 95% confidence interval; a.m.a.s.l.: meters above sea level; PR: prevalence ratio.
Note: bolded estimates are statistically significant at the 0.05 level.
* Model adjusted by sex, age, education level, socioeconomic level, study area, altitude, smoking, alcohol use, body mass index, and study year. When model was stratified by sex (or body mass index), such variable was not included as confounder.

Table S3 Cross-classification between body mass index (BMI) and abdominal obesity and its association with hypertension: crude and adjusted models excluding those with previous hypertension diagnosis.

	**Crude mode RP (95%CI)**	**Adjusted model * RP (95%CI)**
**BMI and abdominal obesity**		
BMI normal, no abdominal obesity	1.00 (Reference)	1.00 (Reference)
BMI normal, abdominal obesity	1.27 (0.92-1.75)	**2.02 (1.45-2.83)**
BMI overweight, no abdominal obesity	**2.24 (2.04-2.46)**	**1.77 (1.61-1.94)**
BMI overweight, abdominal obesity	**1.61 (1.44-1.79)**	**2.29 (2.03-2.57)**
BMI obese, no abdominal obesity	**3.85 (3.32-4.47)**	**2.74 (2.37-3.18)**
BMI obese, abdominal obesity	**3.19 (2.92-3.49)**	**3.33 (3.02-3.66)**
**BMI and abdominal obesity (among women)**		
BMI normal, no abdominal obesity	1.00 (Reference)	1.00 (Reference)
BMI normal, abdominal obesity	**2.25 (1.58-3.21)**	**1.52 (1.05-2.20)**
BMI overweight, no abdominal obesity	1.08 (0.83-1.40)	1.16 (0.88-1.53)
BMI overweight, abdominal obesity	**2.38 (1.98-2.85)**	**1.90 (1.57-2.31)**
BMI obese, no abdominal obesity	**2.24 (1.02-4.94)**	**2.43 (1.04-5.72)**
BMI obese, abdominal obesity	**3.67 (3.09-4.37)**	**2.84 (2.36-3.42)**
BMI normal, no abdominal obesity	1.00 (Reference)	1.00 (Reference)
BMI normal, abdominal obesity	**6.40 (2.35-17.41)**	**5.03 (2.19-11.60)**
BMI overweight, no abdominal obesity	**1.97 (1.79-2.18)**	**1.88 (1.69-2.08)**
BMI overweight, abdominal obesity	**3.28 (2.79-3.85)**	**2.50 (2.12-2.95)**
BMI obese, no abdominal obesity	**2.82 (2.42-3.29)**	**2.77 (2.37-3.23)**
BMI obese, abdominal obesity	**4.06 (3.67-4.50)**	**3.45 (3.09-3.85)**

95%CI: 95% confidence interval; PR: prevalence ratio.

Note: bolded estimates are statistically significant at the 0.05 level.

* Model adjusted by sex, age, education level, socioeconomic level, study area, altitude, smoking, alcohol use, and study year.
